# A Case of Inadvertent Carotid Arterial Cannulation and Its Successful Closure with a Percutaneous Closure Device

**DOI:** 10.7759/cureus.96204

**Published:** 2025-11-06

**Authors:** Umabalan Thirupathy, Prachi Balani, Arvind Kumar Venkataramana Raju, Mohamed Ahmed, Nitish Sharma, Mazen Roumia

**Affiliations:** 1 Department of Internal Medicine, Cheshire Medical Center, Keene, USA; 2 Department of Medicine, Saint Vincent Hospital, Worcester, USA; 3 Department of Cardiology, Saint Vincent Hospital, Worcester, USA

**Keywords:** carotid artery injury, complete heart block, perclose proglide, vascular access, vascular closure

## Abstract

Iatrogenic carotid artery cannulation is a rare but potentially devastating complication of central venous catheterization. We present the case of an 80-year-old male with coronary artery disease who presented with syncope in the setting of complete heart block. Prior to the planned placement of a permanent pacemaker, he underwent temporary transvenous pacemaker insertion via the right internal jugular vein. Despite the use of ultrasound, his right common carotid artery was inadvertently cannulated and dilated. This was only identified after the application of a pressure transducer. As he had a prohibitive risk for open surgical repair, his right common carotid artery was then repaired less invasively with the use of a Perclose Proglide percutaneous closure device (Abbott Vascular, Inc., Santa Clara, CA, USA), leading to a successful repair without any adverse neurological outcome. A temporary pacemaker was eventually placed via the right femoral vein approach. As the patient had an ejection fraction of 35%, he eventually underwent a biventricular implantable cardioverter-defibrillator placement successfully before being discharged home without further complications. To the best of our knowledge, our case report is only the fourth case in the literature to report the off-label use of a suture-mediated closure device to repair inadvertent carotid arterial cannulation with a good outcome.

## Introduction

Central venous catheterization is a ubiquitous procedure in critical care and cardiology. It is, however, not without potentially fatal vascular complications such as arterial puncture or cannulation, or pulmonary complications such as hemothorax or pneumothorax. As our case went on to show, the use of an ultrasound is not a completely foolproof way to avoid arterial punctures [1]. Inadvertent placement of either the guidewire with or without the dilator into an artery can be identified by connecting a pressure transducer to confirm an arterial waveform [2]. This method provides a more accurate assessment than relying on the bright and pulsatile arterial flow, which may not be evident in patients who are hypotensive or hypoxic [3].

The traditional approach to carotid artery puncture with inadvertent cannulation is by open surgical repair. Vascular closure devices (VCD) are an emerging minimally invasive option to open surgical repair, especially in patients with prohibitive surgical risk [4]. One such device is the Perclose ProGlide (Abbott Vascular, Santa Clara, CA, USA) suture-mediated closure system.

To the best of our knowledge, our case report is only the fourth case in the literature to report the off-label use of a suture-mediated closure device to repair inadvertent carotid arterial cannulation with a good outcome. This case report emphasizes the importance of early recognition of a potential arterial injury and critical decision-making in considering potentially less invasive, off-label interventions against open surgical repair as a viable alternative in a patient with prohibitive surgical risk.

## Case presentation

An 80-year-old male patient presented with syncope to the emergency department. He denied any prodromal symptoms prior to his collapse and was followed by rapid and complete spontaneous recovery. A rhythm strip obtained by the emergency medical services showed a complete heart block with a ventricular escape rhythm and intermittent polymorphic non-sustained ventricular tachycardia. His presenting heart rate was 33 beats per minute, with a blood pressure of 150/90 mmHg. There were no significant findings on physical examination.

Past medical history

The patient’s medical history was notable for coronary artery disease with coronary artery bypass graft, aortic stenosis with bioprosthetic aortic valve replacement, a distant history of pulmonary embolism, and essential hypertension. Relevant medication includes low-dose aspirin. 

Investigations

**Figure 1:**
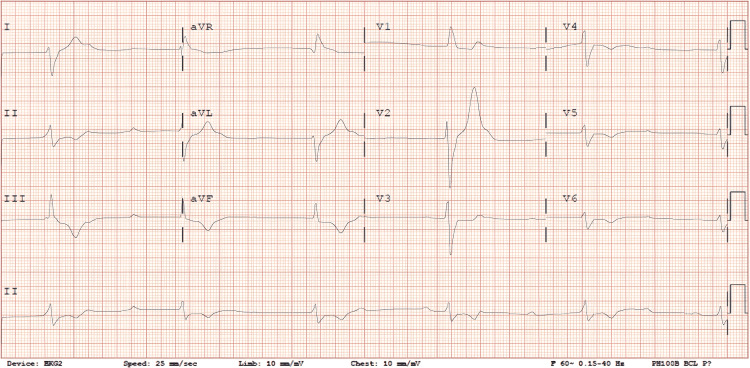
The electrocardiogram on admission showed complete heart block.

The electrocardiogram showed complete heart block with a ventricular escape rhythm (Figure 1). Admission blood counts and relevant electrolytes, such as potassium and magnesium, were unremarkable. Cardiac markers were negative. A decision was made to proceed with temporary venous pacing in the cardiac catheterization lab.

Management

Right internal jugular venous access was attempted under ultrasound guidance. The obtained blood flow was not brisk; hence, a guidewire was inserted before dilation with a 7 French dilator. Application of a pressure transducer to the sheath demonstrated an arterial waveform. Immediate ultrasound examination confirmed the placement of the sheath in the right carotid artery. The sheath had punctured through the right internal jugular vein and landed in the right carotid artery. Vascular surgery was consulted urgently, and the patient was deemed to be high risk for anesthesia and surgical repair; hence, percutaneous closure of the artery was recommended.

**Figure 2:**
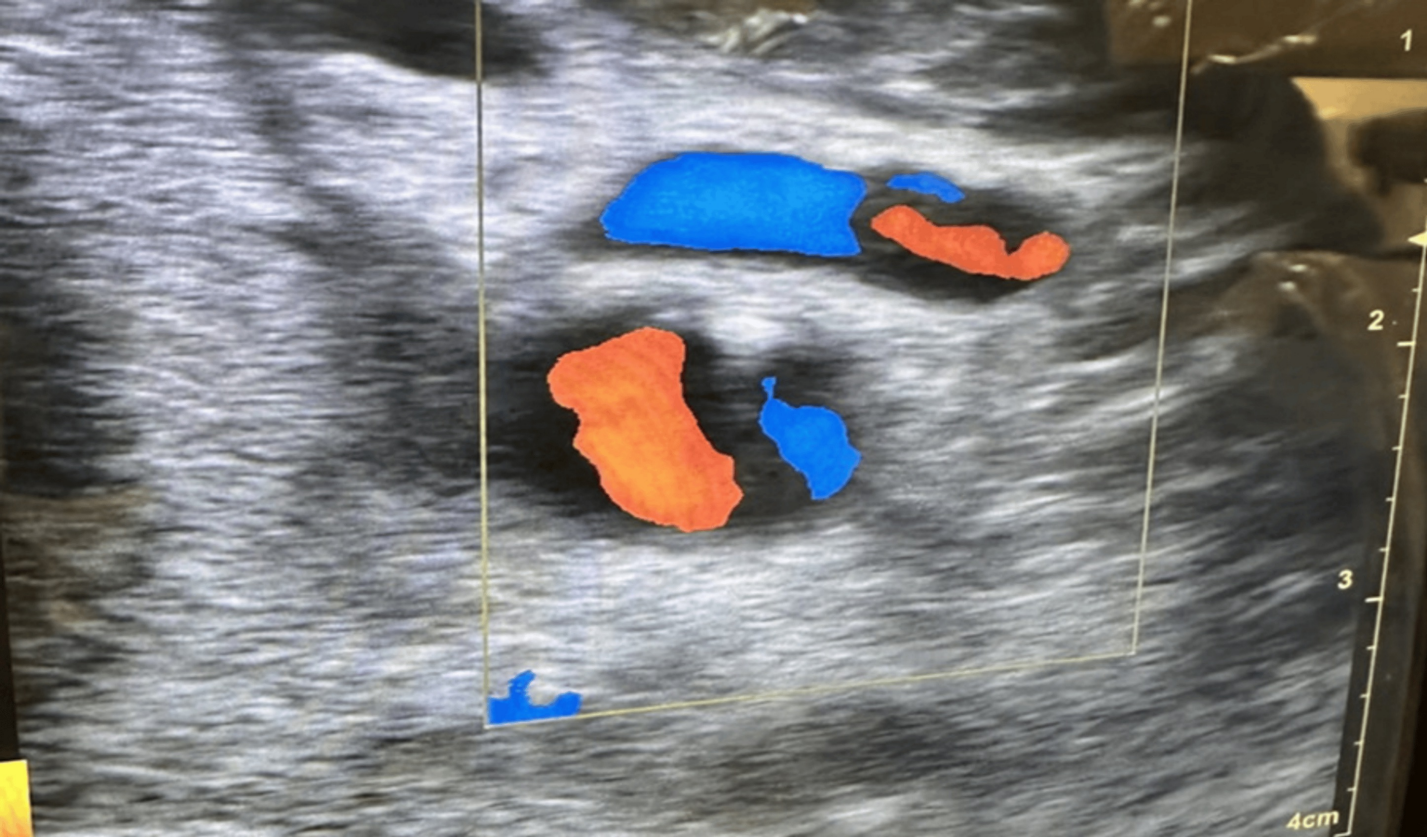
Bedside ultrasound image following arteriotomy closure with the Perclose ProGlide suture device.

The Perclose ProGlide suture-mediated closure device was deployed successfully over the carotid arteriotomy site with suture delivery. Light pressure was applied for 15 minutes without bleeding. A bedside ultrasound performed following the intervention did show robust flow in the carotid artery without evidence of an arteriovenous fistula (Figure 2). As an alternative, a right femoral venous access was utilized for the insertion of the temporary pacing wire with successful pacing. The patient was monitored closely in an intensive care unit setting, with no neurological deficits noted over the next 24 hours. Doppler ultrasound of the carotid arteries bilaterally revealed less than 50% stenosis. An echocardiogram showed severe anteroseptal hypokinesis with an ejection fraction of 35%. The patient underwent successful biventricular implantable cardioverter-defibrillator placement and was discharged home three days later without further complications.

## Discussion

The right internal jugular vein is the preferred choice for venous access due to its reliable anatomy, accessibility, low complication rates, and compatibility with ultrasound [1]. Ultrasound guidance can be used to reduce the rate of complications during central venous catheterizations [2]. Additionally, it can lead to the loss of landmark skills. The incidence of vascular complications during central line placement is usually less than 1%, with femoral access having more frequent rates of complication and subclavian access being the least [3].

The internal jugular vein is preferred for venous access due to its reliable anatomy, easy accessibility, low complication rates, and compatibility with ultrasound [1]. However, carotid arterial puncture is a common complication that occurs in 6.3% to 9.4% of jugular venous catheter placements. If there is a pulsatile or excessive blood backflow, accidental iatrogenic arterial catheterization should be suspected [2]. This can then be quickly managed by application of local pressure.

Failure to detect an arterial puncture can lead to large-bore cannulation (0.1% to 1% incidence), leading to possible complications like stroke, hemothorax, pseudoaneurysms, airway compromise due to hematomas, arteriovenous fistula formation, or death [4, 5]. One way to prevent arterial cannulation is by recognizing arterial punctures through various methods and techniques. These methods vary in reliability and are listed in Table 1 [5].

**Table 1:** Methods of confirming inadvertent arterial puncture and its caveats This table has been adapted from [5].

Methods	Caveats
Pulsatile bright red blood	Unreliable, cannot use alone
Measurement of blood gas for oxygen level	Delay in obtaining results
Ultrasound-guided	Can visualize needle and/or guidewire in the vein more reliably. A guidewire can still puncture into an artery after an initial course in a vein. Less useful for subclavian access
Pressure measurement with column manometry /pressure transducer	Reliable, can be used in conjunction with ultrasound
Fluoroscopy, echocardiography	Can identify location of guide wire, commonly used in interventional radiology, cardiology

The management of arterial injury includes simple manual pressure (if feasible), percutaneous closure devices, open surgery, and endovascular techniques (balloon tamponade, embolization, or covered stent placement) [4].

In instances where it is not practical to perform manual compression, surgical repair is the preferred option for vascular injury repair and to achieve hemostasis. Less invasive alternatives, such as VCDs, balloon tamponade, and covered stent graft placement, have less established safety and efficacy than surgery, especially for sites other than the common femoral artery. High-surgical-risk patients, such as our patient, benefit from these less invasive options.

VCDs are primarily deployed in the repair of femoral arteries but can also be used in other vascular access sites. They significantly reduce the time to hemostasis and improve patient comfort, mobilization, and discharge. In terms of safety and efficacy, VCDs are comparable to manual compression. Two types of VCDs exist: arteriotomy edge-to-edge approximation devices and mechanical plug devices [6].

Numerous VCD devices have been employed, including the Angio-Seal (St. Jude Medical, St. Paul, MN, USA) vascular closure device (26/50), Perclose ProGlide suture-mediated closure system (9/50), StarClose (Abbott Vascular) nitinol clip-mediated closure device (8/50), Prostar XL (Abbott Vascular) device (2/50), ExoSeal (Cordis Corporation, Bridgewater, NJ, USA) closure device (1/50), and several unspecified percutaneous closure devices [4,6].

Suture-based Perclose ProGlide was chosen over open surgical repair as the initial strategy due to the patient's high surgical risk and an access site that was clear of any disease process, as determined by ultrasound. Additionally, a suture-based closure device was chosen over a collagen plug device to maintain the wire position in case of device failure, wherein open surgical repair or other endovascular treatment choices can be explored.

A recent meta-analysis comparing ProGlide and MANTA VCD (Teleflex Incorporated, Wayne, PA, USA) in the repair of trans-femoral access sites did not show a significant difference in rates of adverse outcomes [7]. VCDs may cause complications like bleeding, device failure, thrombosis, dissection, and acute arterial occlusion. However, some studies confirm that percutaneous closure with VCD is safe, and no complications were observed at six, 12, 24, and 48 hours and 90 days post procedure [5].

Direct repair of the resultant arteriotomy via traditional supraclavicular and infraclavicular approaches is a painful, morbid procedure. Minimally invasive techniques should be considered as a replacement [6]. According to a limited observational study by Rey Chaves et al. involving three patients in Latin America, VCDs have up to an 80% success rate in managing these patients, demonstrating excellent safety and efficacy, thereby highlighting their potential as a preferred intervention strategy [8].

## Conclusions

The internal jugular vein is a reliable and easily accessible option for central venous access, but it can lead to complications such as carotid arterial puncture. Various methods exist to detect arterial puncture. For the management of arterial injury, VCDs like Perclose ProGlide offer a safer alternative, reducing time to hemostasis, facilitating earlier mobilization, and reducing the chances of emboli.

Despite the lack of adequate data, VCD use appears to be safe, with no observed issues up to 90 days post-procedure. Minimally invasive techniques like VCDs offer a favorable alternative to traditional direct repair, especially in high-risk patients. These devices have good success rates, making them effective in managing internal jugular vein-related complications.
